# Dermatology

**Published:** 1916-12

**Authors:** Henry Waldo


### Progress of the Medical Sciences.

### DERMATOLOGY.

**Psoriasis** occurs in both sexes and at all ages. The large majority of these cases fall under simple psoriasis, but it is often difficult to decide whether syphilis or seborrhœa may not play a part or better explain it. Some authors regard psoriasis as the extremely dry form of seborrhœa. Supposing one is inclined to the view that the patient is suffering from syphilitic psoriasis, one has to make a differential diagnosis between this condition and pityriasis rosea (Gilbert's disease), and it is possible to confuse a lichen ruber planus with a simple psoriasis. To make the diagnosis between simple psoriasis and some stage of a syphilitic dermatitis, one is guided somewhat by the site selected, the former preferring the limbs and the extensor sides, and is very fond of the elbows, knees, and scalp: the syphilitic rash is more common on the trunk, and the spots are as a rule smaller: the colour, instead of being pink, is usually dark red. Then there is the infiltration test: simple psoriasis is a superficial thing. If a specific eruption is pinched up there is felt to be a distinct deposit under the skin, which is totally absent in simple psoriasis. Again, in nearly all cases of dermatitis depending upon syphilis there are likely to be permanent scars—the white shilling scar; there is never any scarring in simple psoriasis. One is very much helped in differential diagnosis by other well-known signs and symptoms of specific disease. We should not forget that both conditions may be present at the same time.

I have been puzzled very much in deciding between pityriasis rosea and syphilitic psoriasis, and the only safe plan is to wait for a time, for without any treatment, the former often suspicious-looking eruption disappears completely in about six weeks. Do not be tied down exclusively to the skin condition, but look to the glands and other organs.

When a lichen ruber planus becomes generalised, it may be at first perhaps mistaken for psoriasis. But the intense itching of the former stands out from the large majority of cases of psoriasis, whether simple or specific. This form of lichen prefers the flexor sides of the wrists, the neck, and the inner surfaces of the thighs. The characteristic papules are always present, even in extensive cases of general eruption, and would at once settle the diagnosis.

I was once asked to see a young lady with what looked like lupus erythematosus (bat's wing) of the face and across the bridge of the nose. Dr. Radcliffe Crocker saw her, and took exactly the same view. Some years previously she was known to have suffered with simple psoriasis, and after a time this is what it resolved itself into, and was relieved by the ordinary remedies for that condition.

When one feels certain the case is simple psoriasis, it is only fair to tell the patient that it attacks the healthiest people, that it is a harmless rash, and, like seborrhœa capitis, it is incurable permanently, and that the palliative remedies recommended must be continuously persevered with.

A pretty sure cure for psoriasis is to keep the patient in bed, with a daily hot bath with black soap. It is astonishing what a change of air will do in some cases. A middle-aged lady covered with the eruption went away, when it rapidly cleared up, and had not returned twenty years afterwards. Patients frequently notice that simple psoriasis gets well of itself, and then when they least expect it lights up again in all its fury.

I think a lessened nitrogenous diet, with periodical Turkish baths, and daily exercise in the open air with the idea of promoting sweating, keeps off attacks. Arsenic undoubtedly controls chronic psoriasis, if given in gradually increasing doses, and makes recent cases worse. The best internal drug for the latter is salicin, in 15 grain doses three times daily. Salicylic acid ointment, or any other reducing agent, soon gets rid of the scales, but it is unwise to rub it in too vigorously over an extensive surface for fear of absorption. The same applies to all tar applications, and particularly when used for children. There is one serious condition which may develop in connection with simple psoriasis, viz. pityriasis rubra, or dermatitis exfoliativa. It arises from too active external treatment, and chiefly with such remedies as chrysarobin, although if carefully used it is the surest and quickest of all remedies in the cure of psoriasis.

An eruption of the palms and soles is sometimes called psoriasis, but it is usually of an eczematous nature, and is often cured by avoiding soap and water and rubbing in glycerine. If there is much thickening, rub in salicylic acid ointment, and wear loose cotton gloves. For psoriasis of the scalp it is well to rub in a drachm of white precipitate to one ounce of soft soap and vaseline.

Byron Bramwell's plan of administering thyroid extract is quite empirical, and should not be given in out-patient practice unless the patients are being kept in bed.

In a case of psoriasis, whether the scalp is involved or not, do not fail to wash it once or twice a week with soap and water.

N.B—It is now known that the long-continued taking of arsenic conduces to cancer.

HENRY WALDO.

